# Calculating the Mean Amplitude of Glycemic Excursions from Continuous Glucose Data Using an Open-Code Programmable Algorithm Based on the Integer Nonlinear Method

**DOI:** 10.1155/2018/6286893

**Published:** 2018-03-08

**Authors:** Xuefei Yu, Liangzhuo Lin, Jie Shen, Zhi Chen, Jun Jian, Bin Li, Sherman Xuegang Xin

**Affiliations:** ^1^Southern Medical University, Guangzhou, China; ^2^The Third Affiliated Hospital of Southern Medical University, Guangzhou, China; ^3^Guangzhou Power Supply Bureau Co., Ltd., Huadu Branch, Guangzhou, China; ^4^School of Medicine, South China University of Technology, Guangzhou 520006, China

## Abstract

The mean amplitude of glycemic excursions (MAGE) is an essential index for glycemic variability assessment, which is treated as a key reference for blood glucose controlling at clinic. However, the traditional “ruler and pencil” manual method for the calculation of MAGE is time-consuming and prone to error due to the huge data size, making the development of robust computer-aided program an urgent requirement. Although several software products are available instead of manual calculation, poor agreement among them is reported. Therefore, more studies are required in this field. In this paper, we developed a mathematical algorithm based on integer nonlinear programming. Following the proposed mathematical method, an open-code computer program named MAGECAA v1.0 was developed and validated. The results of the statistical analysis indicated that the developed program was robust compared to the manual method. The agreement among the developed program and currently available popular software is satisfied, indicating that the worry about the disagreement among different software products is not necessary. The open-code programmable algorithm is an extra resource for those peers who are interested in the related study on methodology in the future.

## 1. Introduction

Clinical researches have suggested that high glycemic variability may cause more serious damage to the body than high level stable blood glucose [1], which relates to the development of diabetic complication [2–6] and the increase of mortality in critically serious patients without diabetes [7, 8]. In such circumstances, how to quantitatively evaluate the glycemic variability in diabetic blood glucose monitor is essential for the clinical diagnosis and treatment. Several indexes have been proposed for the quantitative evaluation of the glycemic variability, such as MBG, SDBG, IQR, LAGE, M-value, %CV, J-index, IGC, GRADE, MODD, LBGI, HBGI, ADRR, TI, LI, PGS, CONGA, and MAGE [9–16]. Presently, clinicians and researchers trend to choose MAGE as the preferred index [17, 18], making it a “popular standard” in the quantitative evaluation of the within-day glycemic variability.

MAGE is an arithmetic average of either the upward or downward of all glycemic excursions exceeding the threshold (standard deviation of blood glucose (SDBG) obtained from all blood glucose concentrations within 24-hour period); the direction of the calculation is determined by the first countable excursion [19–21]. Following the original definition by Service et al. [19], clinicians and researchers applied the “ruler and pencil” graphical approach to calculate MAGE. However, this kind of manual approach is time-consuming and error-prone, when dealing with huge amount of data typically characterized by 288 observations in 5 minutes apart over a 24-hour period, generated from continuous glucose monitoring (CGM). Thus, the development of computer-aided programs to calculate MAGE becomes an urgently needed task.

To address the urgent need above, several automatic programs have been developed [22–25], making great contribution to the automatic calculation of MAGE. However, it was pointed out that the agreement among them is poor [26]. Therefore, more studies are required to explore the automatic calculation of MAGE. Currently available programs can be roughly divided into two groups. One group has detailed descriptions about algorithm with graphical display, such as the automated algorithm described by Baghurst [22] and the computer software described by Fritzsche et al. (Fritzsche) [23]. The other group only provides executable software for automatic calculation, without detailed descriptions of the algorithms used in the software, such as the web-based application “GlyCulator” [24] and the Excel-based workbook “EasyGV” [25]. However, to the best of our knowledge, all of these methods did not provide programmable open codes, which are important resources required for peers to implement more studies on methodology in this field.

Therefore, in this report, we developed a mathematical algorithm based on integer nonlinear programming method. Following the proposed mathematical method, a computer-aided program named MAGECAA v1.0 was developed. The code of our program is open; if peers are interested in it, please contact xuefeiyu@smu.edu.cn for downloading. To validate the developed program, comparison study was implemented using blood glucose CGM datasets obtained from T1D, T2D, and gestational diabetes patients against the manual method (MAGEo) and other currently popular software products.

## 2. Materials and Methods

### 2.1. The Proposed Mathematical Algorithm

Let *f*(*t*)  (*t* = *t*_1_, *t*_2_,…, *t*_*L*_) represent the discrete blood glucose values obtained in CGM; then *f* is a discrete function defined in time set {*t*_1_, *t*_2_,…, *t*_*L*_}. Let SDBG be the standard deviation of *f*(*t*)  (*t* = *t*_1_, *t*_2_,…, *t*_*L*_). A graph depicting the glycemic variability can be formed by connecting all the discrete values of function *f*. When the difference of a peak and an adjacent nadir exceeds SDBG, the corresponding peak is labeled as a valid peak. The key point for the calculation of MAGE is to correctly count the valid peak or nadir.

Amplitude is the difference of functional values in a peak and a nadir of the function graph. A valid amplitude is labeled and counted when it is bigger than SDBG. To compute MAGE, these valid amplitudes of function *f* should be firstly searched. From the mathematical perspective, a peak or nadir should be an extreme point of the function. These extreme points related to valid amplitudes are called valid extreme points. The MAGE computation problem can be solved by calculating all valid extreme points.

Suppose that {*t*_*l*_1__, *t*_*l*_2__,…, *t*_*l*_*N*__} is the sequence of all extreme points of function *f*. We know that the local maximum points and local minimum points of function *f* should be arranged as follows: interleaved between a maximum point and a minimum point. For simplicity, {*t*_*l*_1__, *t*_*l*_2__,…, *t*_*l*_*N*__} is denoted as {1,2,…, *N*}, and {*n*_1_, *n*_2_,…, *n*_*K*_} is an arbitrary valid subsequence of sequence {1,2,…, *N*}. These local maximum points and local minimum points can be staggered in subsequence {*n*_1_, *n*_2_,…, *n*_*K*_}. Then

(1) 1≤n1<n2<⋯<nK≤N,−1nk+1−nk=−1,k=1,2,…,K−1,

where 2 ≤ *K* ≤ *N*. This equation ensures that the selected extreme points are staggered.

Thus, the above MAGE computation problem can be transformed to an integer nonlinear programming (INLP) problem.

(2) arg maxK,n1,n2,…,nK ZKn1,n2,…,nK=arg maxK,n1,n2,…,nK∑k=1K−1ftnk+1−ftnk,

(3) subject to ftnk+1−ftnk≥SDBG,k=1,2,…,K−1, −1nk+1−nk=−1,k=1,2,…,K−1.

According to the principle of INLP problem, function (2) should have an optimum solution {*n*_1_^*∗*^, *n*_2_^*∗*^,…, *n*_*K*_^*∗*^} and an optimum value *Z*_*K*_^*∗*^ with respect to a constant *K*; it should also have an optimum value *Z*_*K*−1_^*∗*^ for constant *K* − 1, and

(4) $$\begin{array}{c} Z_{K-1}^{*}\leq Z_{K}^{*}. \end{array}$$

Suppose that *K*^*∗*^ is the maximal *K* with respect to the optimal solution of function (2), and then the optimal solution {*n*_1_^*∗*^, *n*_2_^*∗*^,…, *n*_*K*^*∗*^_^*∗*^} represents the valid extreme points to compute MAGE.

If the extreme point *n*_1_^*∗*^ is the local minimum point, then

(5) MAGE+=1K∗/2∑k=1K∗/2fnk−1∗2+2−fnk−1∗2+1.MAGE−=1K∗−1/2∑k=2K∗−1/2fnk−1∗2+1−fnk−1∗2,MAGE=MAGE+,MAGEa=MAGE++MAGE−2,

If the extreme point  *n*_1_^*∗*^ is the local maximum point, then

(6) MAGE+=1K∗−1/2∑k=2K∗−1/2fnk−1∗2+1−fnk−1∗2,MAGE−=1K∗/2∑k=1K∗/2fnk−1∗2+2−fnk−1∗2+1,MAGE=MAGE−,MAGEa=MAGE++MAGE−2.

Now, our object is to get the optimal solution {*n*_1_^*∗*^, *n*_2_^*∗*^,…, *n*_*K*^*∗*^_^*∗*^} of function (2). If the INLP problem is solved by enumeration algorithm, the amounts of different subsequences {*n*_1_, *n*_2_,…, *n*_*K*_} should be $\left(\begin{array}{c} N\\ K \end{array}\right)$. For 3 ≤ *K* ≤ *N*, the amounts of all subsequences are

(7) $$\begin{array}{c} \left(\begin{array}{c} N\\ N \end{array}\right)+\left(\begin{array}{c} N\\ N-1 \end{array}\right)+\cdots +\left(\begin{array}{c} N\\ 3 \end{array}\right)\approx 2^{N}, \end{array}$$

It is difficult to directly solve function (2) when *N* is large. A faster optimization algorithm is usually used to solve the above INLP problem. For example, a penalty function algorithm [27, 28] was used here to transform the above INLP problem (2) to an unconstrained optimization problem as follows.

Set

(8) gkn1,n2,…,nK=SDBG−fnk+1−fnk,hkn1,n2,…,nK=−1nk+1−nk+1,k=1,2,…,K−1.

The new unconstrained integer optimization problem becomes

(9) arg minn1,n2,…,nK YKn1,n2,…,nK=arg minK,n1,n2,…,nK−∑k=1K−1fnk+1−fnk+∑k=1K−1μmax⁡0,gk2+∑k=1K−1λhk2,

where 3 ≤ *K* ≤ *N* and *μ*_*k*_ and *λ*_*k*_  (*k* = 1,…, *K* − 1) are penalty coefficients and tend to be +*∞*.

When the optimum value *Y*_*K*_^*∗*^ > 0, problem (9) has no optimal solution.

Differential evolution (DE) algorithm, a faster optimization algorithm proposed by Storn and Price [29], is a simple but powerful population-based stochastic search technique to solve global optimization problems over continuous domains. Many researchers modified DE algorithm to improve its performance when it was applied to a specific problem [30–33]. The idea of a modified DE algorithm proposed by Lin [34] to solve mixed-integer nonlinear programming problem is used here.

DE searches for a global optimal point in an *n*-dimensional hyperspace. Let  $\bar{S}$  be the *K*-dimensional search space of the INLP problem under consideration. The DE evolves a population of NP  *n*-dimensional individual vectors, that is, solution candidates, $N_{iK}=\left(n_{i1},\ldots ,n_{iK}\right)\in \bar{S}$, *i* = 1, …, NP, from one generation to the next. The evolution begins with a randomly initialized population of *n*-dimensional integer parameter vectors in space {1,2,…, *N*}^*K*^. In each vector, integer parameters are sorted in an ascending order. Each vector forms a candidate solution to the unconstrained optimization problem. At each generation *G*, DE employs the mutation and crossover operations to produce a trial vector **U**_*iK*_^*G*^ for each individual vector **N**_*iK*_^*G*^, also called target vector, in the current population.

The details of the employment of the DE algorithm to solve the above INLP problem are as follows from (a) to (d).

*(a) Initialization.* A randomly initialized population is created to cover the entire search space uniformly as in the following form:

(10) NiK0=1,1,…,1+NINTρi1,ρi2,…,ρiK×N−1,

where *ρ*_*ij*_ is a random number in the range [0,1] and NINT[**B**] is expressed as the nearest integer vector to real vector **B**.

*(b) Mutation Operation.* For each target vector **N**_*iK*_^*G*^ at generation *G*, randomly sample three other individuals **N**_*r*_1_*K*_^*G*^, **N**_*r*_2_*K*_^*G*^, and **N**_*r*_3_*K*_^*G*^ from the same generation, where *r*_1_, *r*_2_, and *r*_3_ are random and mutually different integers generated over the range [1, *NP*], which should be different from the current trial vector's index *i*. Then an associated mutant vector **V**_*iK*_^*G*^ = (*v*_*i*1_^*G*^,…, *v*_*iK*_^*G*^) can be generated by using strategy:

(11) $$\begin{array}{c} V_{iK}^{G}=N_{r_{1}K}^{G}+NINT\left[\;F\times \left(\;N_{r_{2}K}^{G}-N_{r_{3}K}^{G}\;\right)\;\right], \end{array}$$

where *F* is a factor in [0,1] for scaling differential vectors.

*(c) Crossover Operation.* The crossover operation is applied to each pair of the generated mutant vector **V**_*iK*_^*G*^ and its corresponding target vector **N**_*iK*_^*G*^ to generate a trial vector **U**_*iK*_^*G*^ = (*u*_*i*1_^*G*^, *u*_*i*2_^*G*^,…, *u*_*iK*_^*G*^).

(12) uijG=vijG,rand0,1≤CR or j=jrand,nijG,otherwise,j=1,2,…,K,

where CR ∈ [0,1] is a crossover constant that is determined by users. *j*_rand_ is a randomly chosen index in [1, *K*] which ensures that *u*_*ij*_^*G*^ gets at least one parameter from *v*_*ij*_^*G*^. The integer parameters of target vector **N**_*iK*_^*G*^ are also sorted from small to big.

*(d) Selection Operation.* The trial vector **U**_*iK*_^*G*^ is compared to its corresponding target vector **N**_*iK*_^*G*^ using the greedy criterion to decide whether a member of generation *G* + 1 existed or not. If vector **U**_*iK*_^*G*^ yields a smaller cost function value *Y*(**U**_*iK*_^*G*^) than *Y*(**N**_*iK*_^*G*^), then **N**_*iK*_^*G*+1^ is set to **U**_*iK*_^*G*^; otherwise, the old vector is retained. The operation is expressed as follows:

(13) $$\begin{array}{c} N_{iK}^{G+1}=\left\{\begin{array}{cc} U_{iK}^{G}, & \text{if }Y\left(U_{iK}^{G}\right)<Y\left(N_{iK}^{G}\right),\\ N_{iK}^{G}, & \text{otherwise}. \end{array}\right. \end{array}$$

**Figure 1:**
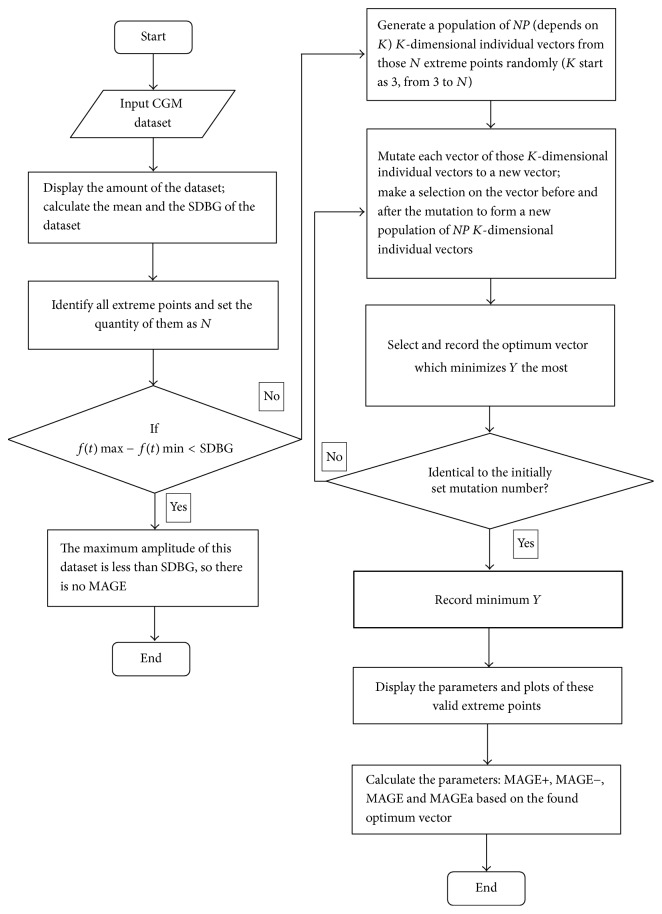
Flow of the algorithm of the program of MAGECAA v1.0.

The above (b) to (d) steps are repeated till the evolution times arrived to certain number (general *G* = 200); from the last evolutionary generation, the individual vector **N**_*iK*_^*G*^ with the smallest value of the objective function *Y* is the optimal solution of the problem of the current *K*-dimensional extreme point combination. The algorithm will search *K* from 3 to *N* till the objective function *Y* obtains the minimum value so as to obtain the optimal extreme point combination {*n*_1_^*∗*^, *n*_2_^*∗*^,…, *n*_*K*^*∗*^_^*∗*^} and then use formulas (5)~(6) to calculate the MAGE value.  Figure 1 shows the entire algorithm.

### 2.2. The Developed Program Based on the Proposed Mathematical Method

**Figure 2:**
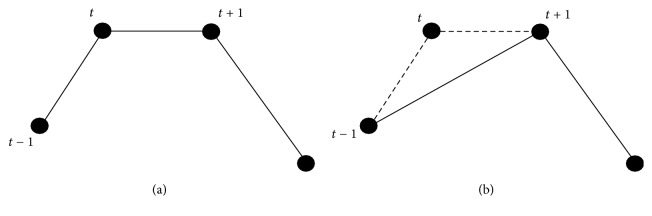
(a) shows turning points *t* and *t* + 1 to be equal in values (not being extreme points) but there is an amplitude from peak point *t* − 1 to nadir point *t*. (b) shows the combination of points *t* and *t* + 1 as one point *t* + 1 or remove point *t*; then let point *t* + 1 be a local maximum point.

Based on the proposed mathematical method, a computer automated program named MAGECAA v1.0 was developed. The MAGE calculation program can be described as a process that selects valid extreme points from a time-ordered set of glucose concentrations whose adjacent differences are all greater than the threshold (typically 1 SDBG obtained from 24-hour period blood glucose concentrations). It can be summarized as finding the optimum vector combination solution of the valid extreme points and using INLP to establish the mathematic method, which can be solved by differential evolution (DE) algorithm. Once all valid extreme points of countable excursions have been identified, the MAGE is determined by MAGE_+_ or by MAGE_−_, depending on the direction of the first countable excursion. For more in-depth understanding, it depends on the first valid extreme point of the vector combination, because that point indicates the direction. In addition, the average of both MAGE_+_ and MAGE_−_, designated as MAGEa, is also calculated. MAGECAA v1.0 is based on INLP and has several different outputs: SDBG, MAGE_+_, MAGE_−_, MAGE, MAGEa, and so forth. Besides it also needs plot to show all valid extreme points joined by straight lines; MATLAB (MathWorks®, USA) is chosen as the programming environment accordingly. Generally, some data points could not be extreme point according to the mathematical definition, like points shown in Figure 2, which shows turning points *t* and *t* + 1 which are equal in values (not being extreme points) but there is an amplitude from peak point *t* − 1 to nadir point *t*, so we combine points *t* and *t* + 1 as one point *t* + 1 or remove point *t* and then let point *t* + 1 be a local maximum point. If the points appear to be opposite, then point *t* + 1 should be a local minimum point.

MAGECAA v1.0 consists of the following major modules: (1) import CGM data and calculate the SDBG as the threshold; (2) identify all extreme points; (3) find the optimum vector combination solution of valid extreme points; and (4) display the calculated parameters and plots.

### 2.3. Collection of CGM Data

The CGM datasets obtained by using CGMS® Gold™ (Medtronic®, USA), collected from clinical treatment, are used to evaluate the proposed program. All CGM datasets were provided by the Third Affiliated Hospital of Southern Medical University. Only complete 24-hour CGM data were selected for comparison study. All patients have provided their written informed consent. 5 CGM recordings from 3 T1D patients, 116 CGM measurements contributed by 58 T2D patients, and 127 CGM measurements based on gestational diabetes patients have been collected. Outpatients had been treated with either diet, oral hypoglycemic agents, oral hypoglycemic agents plus insulin, or insulin alone, depending on their glycemic control.

### 2.4. Data Analysis

The validation of MAGECAA v1.0 was implemented by comparison against MAGEo and MAGEc. A doctor who has been well trained in using the original manual method to analyze CGM data was invited from Department of Endocrinology of the Third Affiliated Hospital of Southern Medical University, and he did not know the effect of MAGECAA v1.0. Our research team analyzed the complete patient population (*n* = 248) CGM measurements using MAGECAA v1.0 and two other popular software products, EasyGV and Fritzsche, whereas the doctor performed a manual analysis on a randomly drawn sample (*n* = 60). As calculator, Fritzsche allows the analyzer to choose whether or not to consider the first and/or the final glucose value of the CGM trace as a start or end point of a glucose excursion; it will result in four different MAGE values per CGM trace. To keep following the original description of the calculation of MAGE and for the comparison of the different software products, the first and final glucose values were both taken into account.

### 2.5. Statistical Analyses

Spearman's correlation analysis was applied in evaluating the relationship between the MAGE values obtained by different methods with respect to the same patients. Bland-Altman plots were used to represent the agreement of the methods [35, 36]. *P* < 0.01 was considered significant.

## 3. Results

**Figure 3:**
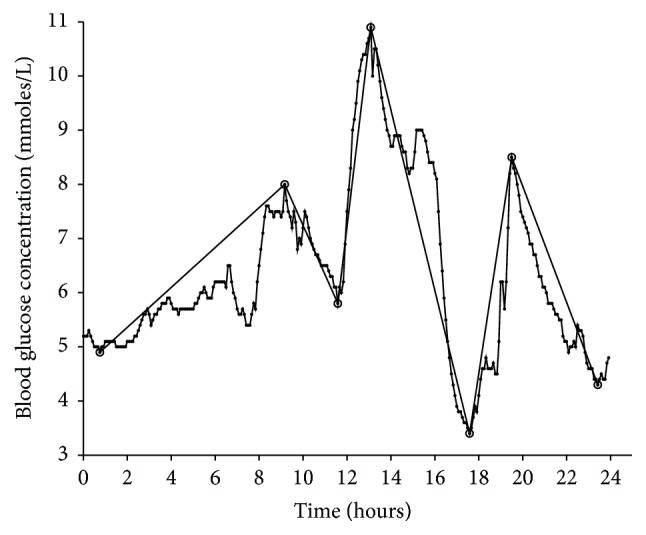
Screenshot of the graphical user interface of the MAGECAA v1.0 based on the MATLAB^®^ programming environment for the mean amplitude of glycemic excursions (MAGE) calculation. After computation, 24-hour continuous glucose monitoring profiles are shown in the plots with all valid extreme points joined by straight lines. Besides the calculation of MAGE, it also calculates the standard deviation of blood glucose (SDBG), the average of all upward valid excursions (MAGE_+_), the average of all downward valid excursions (MAGE_−_), and the average of all valid excursions (MAGEa). The CGM data were collected from patients with type 1 diabetes. After calculation, the results are as follows: SDBG = 1.63 mmoles/L, MAGE_+_ = 4.43 mmoles/L, MAGE_−_ = 4.63 mmoles/L, MAGEc = 4.43 mmoles/L (the first account excursion is from nadir to peak), and MAGEa = 4.53 mmoles/L.

Figure 3 presents the screenshot of the graphical user interface of the developed software named MAGECAA v1.0.

### 3.1. Comparison of MAGECAA v1.0 with the Original Manual Method

**Figure 4:**
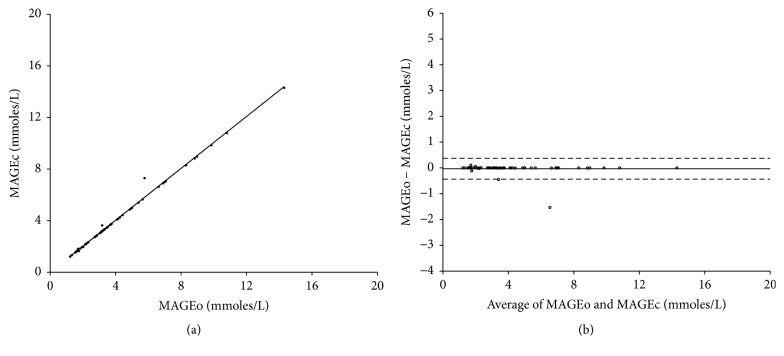
(a) Correlation of the mean amplitude of glycemic excursions values (MAGE) obtained from the proposed program (MAGEc) and the original manual methods (MAGEo). The data of 60 continuous glucose monitoring (CGM) measurements were randomly chosen from all collected CGM data. (b) Bland-Altman plot shows the difference between MAGEc and MAGEo on *y*-axis and the mean of the two computed indices on *x*-axis. The dashed lines represent the 95% confidence limit of the differences between the two methods, and the solid line indicates that the mean difference between the methods is close to 0.

As shown in Figure 4, Spearman's correlation analysis identified that there is a highly significant linear correlation between MAGEc and MAGEo (*r* = 0.998, resp.; *P* < 0.01), and the mean difference found in Bland-Altman plot was −0.03 ± 0.21, which was statistically significantly small. This result indicated that the developed program was robust compared with the manual method.

### 3.2. Agreement of MAGECAA v1.0 with Fritzsche and EasyGV

**Figure 5:**
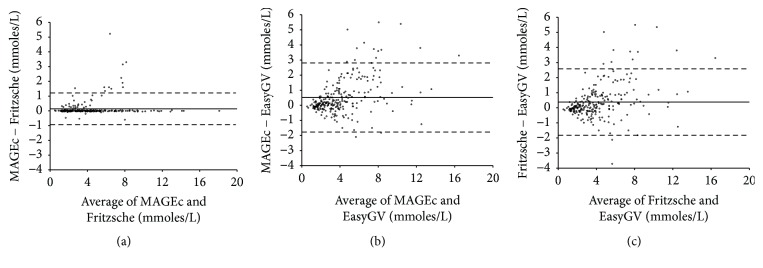
Bland-Altman plots showing the mean difference between MAGEc, EasyGV, and Fritzsche when applied to the same continuous glucose monitoring datasets.

To evaluate the agreement of MAGECAA v1.0 with two currently available popular software products, that is, Fritzsche and EasyGV, we did pairwise comparison of MAGE among them.  Table 1 shows the correlation coefficients for MAGE calculation between the calculators, which ranged from 0.926 to 0.987 (*P* < 0.01 for all).  Figure 5 shows the Bland-Altman plot among the three software products; the dashed lines represent the 95% confidence limit of the differences between the two methods, and the solid line demonstrates that the mean difference between the methods is close to 0, indicating that little difference existed among them. The mean differences are 0.14 ± 0.55 (MAGEc versus Fritzsche), 0.52 ± 1.17  (MAGEc versus EasyGV), and 0.38 ± 1.12  (Fritzsche versus EasyGV), showing the good agreement between these three software products.

### 3.3. Comparison of MAGEc with MAGEa

In the original definition of MAGE, its direction of the calculation is determined by the first countable excursion. So MAGEc = MAGE+ or MAGEc = MAGE−; it is somewhat arbitrary and ignores half of the valid excursions. However, MAGEa represents the average of MAGE+ and MAGE−; it does not consider the direction, thus involving all the valid excursions.

**Figure 6:**
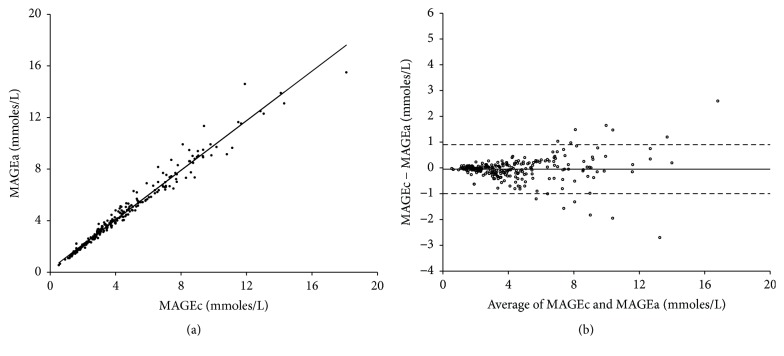
(a) Correlation between the mean amplitudes of glycemic excursions computerized (MAGEc) and the average of all valid glycemic excursions (MAGEa) for the total collected 248 continuous glucose monitoring measurements. (b) Bland-Altman plot shows the mean difference between MAGEc and MAGEa.

To explore whether MAGEa may be a more useful index than MAGEc which depends on MAGE_+_ or MAGE_−_, the relationship between MAGEc and MAGEa was tested by using all the 248 CGM measurements via Spearman's correlation analysis and Bland-Altman analysis. As shown in Figure 6, the correlation coefficient was *r* = 0.993  (*P* < 0.01) and the mean difference was −0.05 ± 0.48. The results showed that little difference was observed between using MAGEa and MAGEc, indicating that MAGEa may be a useful index for evaluating glycemic variability.

## 4. Discussions

We developed a computer-aided open-code program named MAGECAA v1.0 based on INLP algorithm for automatic calculation of MAGE. Compared with the existing methods, the proposed novel method turns to search the optimal solution of the combination of extreme points from overall CGM measurements instead of searching adjacent extreme points from the beginning to the end step by step. As for the computational time, if used for one person, the proposed method is comparable with currently available methods; if used for batch calculation, the proposed method is more powerful. The programmable open codes are useful for the study of methodology for automatic calculation of MAGE. The comparison study using MAGECAA v1.0 against manual method indicated that the agreement is satisfied. The pairwise comparison study between MAGECAA v1.0 and two other available software products, Fritzsche and EasyGV, based on Spearman's correlation analysis and Bland-Altman plots, demonstrated that the agreements between them met the requirement. Our study showed that the worry about the disagreement among the currently available popular software products is not necessary, which is different from the proposal by Sechterberger et al. [26]. The reason may be the increased amount of CGM data used in our research.

The MAGE value depends on MAGE_+_ or MAGE_−_, following the direction of the first accountable glucose excursion. Considering the fact that only one direction of glucose excursion is adopted in current popular software, unavoidably resulting in the omission of the other directions of valid excursions, we implemented an extra experiment in which data from both directions of the valid glycemic excursions are utilized. MAGEa was used to represent the mean of MAGE_+_ and MAGE_−_ for the calculation of MAGE. As shown by our data, a close linear correlation between MAGEa and MAGEc was observed, indicating that the difference between MAGE_+_ and MAGE_−_ is significantly small. Therefore, we proposed that MAGEa might be another suitable parameter to quantify glycemic variability.

To conclude, an open-code software program named MAGECAA v1.0 for automatic calculation of MAGE based on a mathematical algorithm has been proposed and evaluated. The programmable open codes are useful for further methodology study in the future. The comparison study indicated that the agreement among the proposed software and existing software is satisfied, and the worry about the disagreement among currently available different popular software products is not necessary.

## Figures and Tables

**Table 1:** The correlation coefficient and difference of the results separately calculated using MAGEc, Fritzsche, and EasyGV software.

	MAGEc		Fritzsche	
	*r*	mean ± SD	*r*	mean ± SD
		(mmoles/L^−1^)		(mmoles/L^−1^)
MAGEc	—	—	—	—
Fritzsche	0.987	0.14 ± 0.55	—	—
EasyGV	0.926	0.52 ± 1.17	0.926	0.38 ± 1.12

*Note.*  *P* < 0.01.

## Abbreviations

MAGE: Mean amplitude of glycemic excursions
SDBG: Standard deviation of blood glucose
CGM: Continuous glucose monitoring
Fritzsche: Fritzsche et al.'s software
INLP: Integer nonlinear programming
MAGEo: Calculated values obtained from the original manual method
MAGEc: Calculated values obtained from MAGECAA v1.0
MAGE+: The average of all upward valid glycemic excursions
MAGE−: The average of all downward valid glycemic excursions
MAGEa: The average of all downward validated glycemic excursions
DE: Differential evolution.
